# The immediate impact of transcranial magnetic stimulation on brain structure: Short-term neuroplasticity following one session of cTBS

**DOI:** 10.1016/j.neuroimage.2021.118375

**Published:** 2021-10-15

**Authors:** JeYoung Jung, Matthew A. Lambon Ralph

**Affiliations:** a School of Psychology, University of Nottingham, University Park, Nottingham, NG7 2RD UK; b MRC Cognition and Brain Science Unit (CBU), University of Cambridge, UK

**Keywords:** Structural plasticity, Theta burst stimulation, Voxel-based morphometry, Anterior temporal lobe, cTBS combined fMRI

## Abstract

Recent evidence demonstrates that activation-dependent neuroplasticity on a structural level can occur in a short time (2 hour or less) in the human brain. However, the exact time scale of structural plasticity in the human brain remains unclear. Using voxel-based morphometry (VBM), we investigated changes in grey matter (GM) after one session of continuous theta-burst stimulation (cTBS) delivered to the anterior temporal lobe (ATL). Twenty-five participants received cTBS over the left ATL or the occipital pole as a control site outside of the scanner, followed by structural and functional imaging. During functional imaging, participants performed a semantic association task and a number judgment task as a control task. VBM results revealed decreased GM in the left ATL and right cerebellum after the ATL stimulation compared to the control stimulation. In addition, cTBS over the left ATL induced slower semantic reaction times, reduced regional activity at the target site, and altered functional connectivity between the left and right ATL during semantic processing. Furthermore, the decreased ATL GM density was associated with the interhemispheric ATL-connectivity changes after the ATL stimulation. These results demonstrate that structural alterations caused by one session of cTBS are mirrored in the functional reorganizations in the semantic representation system, showing the rapid dynamics of cortical plasticity. Our findings support fast adapting neuronal plasticity such as synaptic morphology changes. Our results suggest that TBS is able to produce powerful changes in regional synaptic activity in the adult human brain.

## 1 Introduction

Neuroplasticity refers to the brain's ability to reorganize itself in response to environmental changes and involves a complex, multilevel process including molecular, synaptic, electrophysiological and structural organization. The scope of neuroplasticity encompasses functional forms including short-term weakening and strengthening of existing synapses through long-term potentiation (LTP) and long-term depression (LTD) and structural types such as synaptogenesis, gliogenesis, and neurogenesis (Bruel-Jungerman et al., 2007; Butz et al., 2009; Holtmaat and Svoboda, 2009). Although traditional neuroscience research has focused on functional forms of neuroplasticity in the adult brain, structural types of plasticity play a critical role in adaptation to environmental changes and diseases such as stroke (Pekna et al., 2012).

Repetitive transcranial magnetic stimulation (rTMS) is a non-invasive brain stimulation technique that alters cortical excitability via changes in synaptic strength (e.g., LTP and LDT), and generates neuroplasticity in the brain (Pell et al., 2011). Since the development of TMS in the early 1980s, rTMS has been extensively used to study the brain-behavioural relationship. It has also been used to induce both structural and functional neuroplasticity as a potential therapeutic tool for depression, schizophrenia, dementia, and stroke (Lefaucheur et al., 2014). Theta-burst transcranial magnetic stimulation (TBS), as an effective rTMS protocol, modulates cortical excitability within a short period of stimulation (less than 5 mins) (Huang et al., 2005). TBS has increasingly and successfully been used to explore the mechanisms and consequences of functional plasticity in the human cortex (Agnew et al., 2018; Hartwigsen et al., 2013; Jung and Lambon Ralph, 2016; Valchev et al., 2016). Animal studies demonstrated that TBS can induce immediate and prolonged functional and structural plasticity (for a review, see Funke and Benali, 2011). TBS modulates the GABA-synthesizing enzymes, presynaptic GABA transporters, and cortical inhibitory interneurons (Funke and Benali, 2010; Trippe et al., 2009). Specifically, continuous TBS (cTBS) has been found to reduce the number of calbindin expressing interneurons, whereas intermittent TBS (iTBS) decreased in parvalbumin expressing cells right after the stimulation (Benali et al., 2011). In the human brain, rTMS/TBS induced morphological changes were observed in grey matter (GM) and white matter (WM) (Allendorfer et al., 2012; Lehner et al., 2014; May et al., 2007). Allendorfer et al. (2012) delivered 10 days of iTBS treatments for aphasic patients and found increased white matter integrity near the prefrontal gyri and in the anterior corpus callosum. May et al. (2007) used voxel-based morphometry (VBM) to detect structural alterations following 5 days of 1 Hz rTMS over the left superior temporal gyrus (STG). They reported increased GM in the targeted area as well as a transient increase and decrease of GM in the contralateral region. Following this study, Lehner et al. (2014) tested 1 Hz rTMS treatment (10 days), targeting the left STG in patients with tinnitus and found decreased GM volume in the bilateral insular and inferior frontal cortex. Contrary to animal studies, three human studies with several sessions of daily rTMS/TBS have reported inconsistent rTMS/TBS-related structural changes in various regions, including the target region as well as remote areas. Yet, as far as we are aware, there has been no study of the immediate effect of rTMS/TBS on the human brain structure, in vivo.

Structural neuroplasticity is thought to be slower and less common than functional plasticity (Bruel-Jungerman et al., 2007). However, the timescale of structural plasticity is still poorly understood. Animal studies have showed that neurogenesis occurs within days, whereas local morphological changes such as formation of new synapses and dendrites can arise on shorter time period, and less than a day (Bruel-Jungerman et al., 2007; Butz et al., 2009; Holtmaat and Svoboda, 2009; Trachtenberg et al., 2002). One study showed that regional structural changes (increased dendrite spines) in the rodent's brain occurred immediately after short-term training (about 1 hour) (Xu et al., 2009). In the human, a long period of training (weeks or months) induces such structural changes (Draganski et al., 2004; Zatorre et al., 2012). In human adults, Sagi et al. (2012) demonstrated that only 2 hours of learning resulted in brain structural alterations using diffusion tensor imaging. Participants in the learning group performed a spatial learning and memory task for 90 min on average. Microstructural changes of hippocampus and parahippocampus were observed, with improved task performance in the learning group compared to the control group (no learning). Their findings were replicated in a subsequent rat study which showed that there was structural remodelling of the rat hippocampus following 2 hour in a water maze task (Sagi et al., 2012). These studies provide evidence that structural plasticity can occur short time scales (~ hours) in the adult brain. It is important to understand to what extent the structural plasticity arises in the adult brain following environment demands and disease because this type of cortical plasticity is associated with short-and long-term therapeutic effects.

Focusing on the issue of the timescale of structural plasticity, we used one session of TBS to explore its immediate effect of the brain structure. Previously, we showed that cTBS over the anterior temporal lobe (ATL) induced rapid, adaptive functional reorganization in the semantic representation system, revealing regional activity changes at the target region and connected homologues region as well as altered functional connectivity between them (Jung and Lambon Ralph, 2016). Here, we re-analysed the data set from our previous study with respect to structural plasticity induced by cTBS, a question that we did not address in the original publication. Healthy subjects received cTBS over left ATL and the occipital pole (Oz) as a control stimulation (Jung and Lambon Ralph, 2016). After the stimulation, structural images were obtained and analysed using VBM to assess changes in the GM and WM. Given the evidence from animal studies and human learning studies that structural changes can occur shorter time scales (e.g., one session of TBS in rats or 2 hours training in both rats and human), we hypothesised that one session of cTBS might alter brain structure, which can be captured by in vivo neuroimaging. Specifically, animal studies with the inhibitory rTMS/TBS protocol have reported acute effects of stimulation, such as decreased dendritic integration and the reduction of the number of interneurons (Benali et al., 2011; Funke and Benali, 2011). Here, we hypothesized that one session cTBS over the ATL would decrease the GM density at the target region as well as the related white matter tract. In addition, we predicted that the structural alterations induced by cTBS would be associated with short-term functional neuroplasticity in the semantic representation system as observed in the parallel task fMRI.

## 2 Materials and methods

### 2.1 Subjects

All data have previously been included in a previous publication (Jung and Lambon Ralph, 2016). We re-analysed the MRI data set with respect to microstructural changes in grey and white matter caused by cTBS. Accordingly, data from 25 healthy, right-handed native English speakers were included (7 males, mean age, 21.9 ± 3.7 years, range from 19 to 34 years). Data from two participants were excluded in the analysis due to excessive head movements during fMRI (over a voxel). Handedness was assessed using the Edinburgh Handedness Inventory (Oldfield, 1971). All subjects provided informed written consent. The study was approved by the local ethics committee.

### 2.2 Experimental design and procedure

**Fig. 1:**
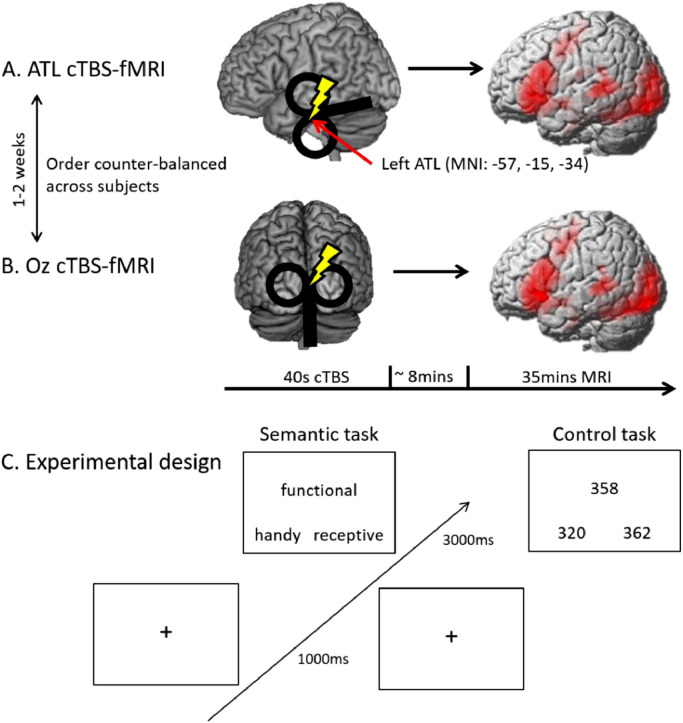
Experimental design and procedures. A) cTBS over the left ATL and following fMRI with tasks. B) cTBS over the occipital pole (Oz) as a control stimulation. C) Experimental design.

A detailed description of the procedure has been previously published (Jung and Lambon Ralph, 2016). We here summarize the important steps. We used a within-subject design to test the effects of one-session cTBS on grey and white matter density. Each subject participated in two MRI sessions (Fig. 1). In each session, cTBS was applied prior to the MRI outside of the scanner: the left ATL stimulation and Oz stimulation as a control site. Sessions were separated by at least one week to avoid carry-over effects. The order of ATL and Oz cTBS was counterbalanced between subjects.

In the MRI, all participants performed a semantic judgement task and number judgement as a control task (Fig. 1C). In the semantic task, subjects saw three words on the screen and select which of two words (bottom) was more related to a target word (top) in meaning. In the control task, subjects saw three numbers and chose which of two numbers (bottom) was closer to the target number (target) in numerical value. Each trial started with 1 s fixation followed by the stimuli presented for a fixed duration of 3 s. A block design fMRI was used with three condition blocks: semantic, control and fixation. Each task block had four trials of a task and the fixation blocks (8 s) were interleaved between task blocks. E-prime software (Psychology Software Tools Inc. Pittsburgh, PA, USA) was used to display stimuli and to record responses.

### 2.3 Theta-burst stimulation

cTBS (600 pulses at 50 Hz for 40 s) was delivered over the stimulation sites using a Magstim SuperRapid 2 with a figure-of-eight coil (70 mm standard coil, MagStim Company, Whitland, UK) according to Huang et al. (Huang et al., 2005). cTBS was applied during the ATL and Oz at 80% of the resting motor threshold (RMT). The mean of stimulation intensity was 49.1% ranging from 36% to 61%.

All subjects were scanned to obtain a high-resolution T1-weighted anatomical image (in-plane resolution of 1 mm and a slice thickness of 1.8 mm with an acquisition matrix 256 × 256 voxels) using a 3T Philips MR Achieva scanner prior to the experiment.

The target ATL site was based on a previous distortion-corrected fMRI study (Visser et al., 2012) [MNI: −57, −15, −34]. The ATL coordinate was transformed into each subject's native space by normalizing each subject's MRI scan against the MNI template using Statistical Parametric Mapping software (SPM8, Wellcome Trust Centre for Neuroimaging, London, UK). Then, the inverse of each resulting transformation was used to convert the target MNI coordinate to the untransformed individual naïve space coordinate. These ATL coordinates were used to guide the frameless stereotaxy, Brainsight TMS-MRI co-registration system (Rogue Research, Montreal, Canada). The control site was the occipital pole (Oz) localised by international 10–20 system.

### 2.4 Image acquisition

MRI was performed on a 3T Philips Achieva scanner using an 8-element head coil with a SENSE factor of 2.5. A high-resolution T1-weighted image was acquired using a 3D MPRAGE pulse sequence with 200 slices, in plane resolution 0.94 × 0.94 mm, slice thickness 0.9 mm, TR = 8.4 ms, TE = 3.9 ms. For fMRI, a dual-echo protocol developed by Halai et al. (Halai et al., 2014) was used in order to compensate the signal dropout around rostral temporal areas (42 slices, 96 × 96 matric, 240 × 240 × 126 mm FOV, in-plane resolution 2.5 × 2.5 mm, slice thickness 3 mm, TR = 2.8 s, TE = 12 ms and 35 ms).

### 2.5 Voxel-based morphometry

Voxel-based morphometry (VBM) was used to investigate the GM and WM changes, via the VBM8 toolbox (http://dbm,neuro.uni-jena.de/vbm8) in SPM8. Preprocessing of the data involved normalization, segmentation, modulation, and smoothing (Ashburner and Friston, 2000). We created a customized GM and WM templates from subjects in this study (Oz stimulation secession). Normalization parameters were estimated using an optimized protocol in order to facilitate optimal segmentation by normalizing extracted GM and WM images to the customized GM and WM templates (Good et al., 2001). Then the optimized parameters were reapplied to the original brain images. The images were aligned with the MNI space, corrected for non-uniformities in signal intensity and partitioned into GM, WM, cerebrospinal fluid (CSF), and background. Modulation was performed for volume change correction by modulating each voxel with the Jacobian determinants derived from the spatial normalization, which allows us to test regional differences in the absolute amount of GM and WM (Ashburner and Friston, 2000). Finally, all images were smoothed by convolving them with an isotropic Gaussian kernel of 8 mm full-width at half maximum.

Voxel-by-voxel statistical procedures using the GLM were performed to examine regional specific GM and WM changes between ATL and Oz stimulation. The random effects model performed paired t-tests for each subject's scans using TMS sites (ATL vs. Oz), accounting for the total intracranial volume. The statistical threshold set for all contrasts was *p* < 0.001 uncorrected with an extent threshold of 100 contiguous voxels. We hypothesized that cTBS over the left ATL would alter the brain morphology in this region and related white matter tract based on the previous findings (Jung and Lambon Ralph, 2016). A priori region was defined as an 8 mm sphere in the left ventral ATL (vATL MNI: −33, −9 −39). For the white matter tract, we used the anterior commissure map from NatBrainLab (http://www.natbrainlab.co.uk/atlas-maps). We applied a threshold of FWE-corrected *p* < 0.05 for the multiple comparisons correction after a small volume correction (SVC), using a priori region.

### 2.6 fMRI analysis

A detailed description of the fMRI analysis has been previously published (Jung and Lambon Ralph, 2016). We summarize the important steps, here. The dual gradient echo images were extracted and combined using in-house Matlab scripts (Halai et al., 2014). The combined images were realigned, coregistered, normalized to the structural image and smoothed with an 8 mm full-width half-maximum Gaussian filter using SPM8. General linear model (GLM) analysis was performed to set up a fixed-effect model with each task condition (semantic and control) and to assess differences in activation between the contrasts (semantic > control) using a random-effect model. Region of interest (ROI) analysis was performed using a priori ROI (vATL).

Dynamic causal modelling (DCM) was used to estimate effective connectivity between the bilateral ventral ATL after suppression of the left ATL by cTBS. DCM estimates and makes directional inferences in a predefined set of brain regions in different experimental contexts (Friston et al., 2003). The DCM models were based on a bilateral ATL network which showed significant TMS effects in previous analysis (Jung and Lambon Ralph, 2016). Thus, the model consisted of bilateral, intrinsic connections between the ventral ATLs. Then, we set up three possible modulatory connections between them, reflecting the changes in the intrinsic connections induced by the experimental conditions (ATL stimulation in this study). Bayesian model selection (Stephan et al., 2009) was applied to determine which DCM models were the most likely given the observed fMRI data. The result showed the winning model having a connection from the left to right ATL during semantic processing. We extracted individual specific parameters of the winning model, including the intrinsic connectivity (left ventral ATL → right ventral ATL and right ventral ATL → left ventral ATL) and the modulatory connectivity (left ventral ATL → right ventral ATL) and explored the relationship between the connectivity and brain morphological changes induced by cTBS (Pearson's correlation, *p* < 0.05).

## 3 Results

A detailed description of the results has been previously published (Jung and Lambon Ralph, 2016). Here, we summarize the key findings from the previous study and also report the new findings related to changes in the GM and WM induced by cTBS.

Behavioural results demonstrated inhibitory cTBS effects (slower reaction time; RT) after the ATL stimulation compared to the control stimulation during the semantic task. In the fMRI results, cTBS over the left ATL induced decreased task-induced activation in the left ATL as well as a compensatory up-regulation in the homologue right ATL. Furthermore, the effective connectivity between the ATLs was modulated by cTBS, showing a compensatory facilitation from the right ATL (intact region) to the left ATL (lesioned region) and increased task-specific connectivity during semantic processing (left ATL → right ATL) (see the summary in Table S1). These results demonstrated fast, adaptive functional reorganization of semantic system after one session of cTBS intervention.

**Fig. 2:**
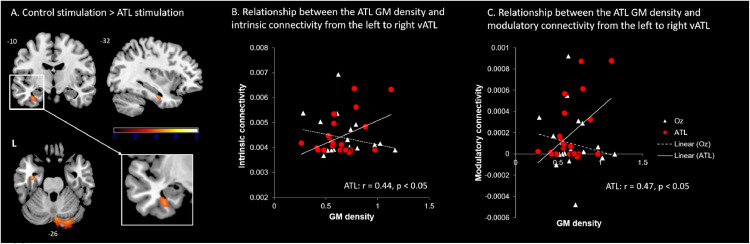
The GM changes following cTBS over the left ATL compared to the control stimulation. A) Significantly decreased GM at the left ventral ATL and right cerebellum. B) The ATL GM density was positively correlated with intrinsic connectivity between the ATLs only after the ATL stimulation. C) The ATL GM density following the ATL stimulation was positively correlated with modulatory connectivity between the ATLs during semantic processing. Red circle represents the ATL stimulation. White diamond represents the control stimulation.

VBM results showed that there was a significant transient decrease in the left ventral ATL volume (MNI: −36 −12 −24, cluster size = 108, p _SVC-FWE corrected_ = 0.005) after the ATL stimulation compared to the control stimulation (Fig. 2A). We also found a significant GM decrease in the right cerebellum (MNI: 17, −37, −48, cluster size = 1269, MNI: 5, −76, −26, cluster size = 1065, p _FWE-corrected_ < 0.001). There was no GM changes in the comparison of ATL stimulation > Oz stimulation. The dynamic changes of the GM density was specific to the ATL stimulation. Then, we investigated the relationship between the GM density after the stimulation and functional short-term plasticity in the semantic system - effective connectivity between the ATLs. We found that there was a significant positive correlation between the ATL GM density and ATL-connectivity only after the ATL stimulation. Participants with greater GM in the left ventral ATL showed stronger connectivity between the left and right ATL (intrinsic connectivity: *r* = 0.44, *p* = 0.017, modulatory connectivity: *r* = 0.47, *p* = 0.012) (Fig. 2B & C). There was no significant correlation between the ATL GM density and ATL-connectivity after the control stimulation (intrinsic connectivity: *r* = −0.23, *p* = 0.16, modulatory connectivity: *r* = 0.01, *p* = 0.49). Additionally, we examined the GM density in the target site, the ventrolateral ATL (MNI: −57, −15, −34), after the stimulation. We found reduced GM density after the ATL stimulation compared to the control stimulation and the residual GM density after the ATL stimulation was also positively correlated with the ATL-interhemispheric connectivity (Fig. S1). It is noted that there was no significant changes in occipital cortex following the Oz stimulation (control stimulation). No white matter changes were detected.

## 4 Discussion

Our results suggest that structural alterations in GM can occur very rapidly with one session of cTBS. Our results correspond to the time scale of TMS-induced structural plasticity in animal models (Funke and Benali, 2011) and learning-related morphological alterations in the human brain (Sagi et al., 2012). Importantly, these local structural alterations were associated with parallel functional connectivity changes in the targeted system, following cTBS. The local structural plasticity found in the current study support the suggestion that TBS is able to produce changes in regional synaptic activity in the adult human brain (Siebner and Rothwell, 2003; Thomson et al., 2020). Our surprising results suggest a fast adaptive neural system, supporting previous evidence of dynamic semantic processing (Jung and Lambon Ralph, 2016). The results also contribute to our understanding of structural plasticity involved in clinical intervention with rTMS/TBS.

Structural alterations induced by TMS are not well defined in the human brain but animal studies have demonstrated that rTMS/TBS induce immediate and prolonged structural plasticity in excitatory and inhibitory synapses (e.g., changes in size and number of related cells or receptors), concomitant with functional plasticity (e.g., pre- and postsynaptic activity, neurotransmitter release) (Funke and Benali, 2011; Lenz et al., 2016). Recently, Thomson et al. (2020) investigated the molecular mechanisms of TBS in living human neurons. They reported an increased expression of plasticity genes following one-session of iTBS (600 pulses), compared to sham stimulation. Employing an in vitro human neuron-like model, they identified several gene expression changes supporting iTBS-induced plasticity. These studies provide strong evidence of the immediate effects of TBS in structural plasticity. Here, we demonstrated that cTBS, an inhibitory TBS, reduced the GM density at the targeted cortical region in vivo. VBM detects differences in local concentration or volume of GM and WM per voxel, changes in the classification of individual voxels, and potentially a combination of both (Ashburner and Friston, 2000). The underlying mechanisms of GM changes detected by VBM include axon sprouting, dendritic branching and synaptogenesis, neurogenesis, changes in glia number and morphology, and even angiogenesis (Zatorre et al., 2012). Although the GM changes we observed might reflect alterations in cell genesis/loss, the time scale of our study corresponds to fast adapting neuronal plasticity such as synaptic morphology changes (e.g., decreased dendritic integration and the reduction of cell size or the number of interneurons) (Funke and Benali, 2011; Lenz et al., 2016), rather than slow mechanisms as neuronal or glia cell genesis/loss (Kempermann et al., 1997).

Studies using rTMS/TBS with functional imaging have demonstrated that unilateral rTMS/TBS leads to bilateral changes at a functional level, reflecting functional connectivity alterations between the stimulated region and functionally-connected remote homologous areas in the contralateral hemisphere in various cognitive domains (Agnew et al., 2018; Andoh and Paus, 2011; Binney and Lambon Ralph, 2015; Hartwigsen et al., 2013; Jung and Lambon Ralph, 2016; Lee et al., 2003; O'Shea et al., 2007; Sack et al., 2005; Valchev et al., 2016). These studies suggest that rTMS/TBS can induce a compensatory short-term functional reorganization with an increased contribution from the homologous area in the contralateral hemisphere after transient disruption by inhibitory stimulation. Consistent with these studies, May et al. (2007) showed that 5 days of iTBS over the left superior temporal gyrus induced increase and decrease GM volume at the bilateral superior temporal gyrus. Although we did not observe structural changes in the homologous right ATL following cTBS over the left ATL, our results showed that cTBS decreased GM density and regional activity in the left ATL as well as changes in functional connectivity between the targeted ATL and the homologous ATL in the contralateral hemisphere. Importantly, the GM alterations at the target region were associated with functional connectivity changes in the targeted system (bilateral ATLs) during semantic processing. Our findings suggest that TBS-induced structural neuroplasticity co-occurs with changes in functional processing and provide important insights about dynamic, fast-adaptive neural systems (Siebner and Rothwell, 2003). Furthermore, these findings provide empirical evidence supporting the theoretical framework that structural forms of plasticity serve an important function in processing information in neural systems when the flexible networks face information demands (Chambers et al., 2004).

In addition to the GM alterations in the left ATL following cTBS, we also observed GM reduction in the right cerebellum. It has been suggested that rTMS/TBS can influence remote brain regions by modulating the activity of interconnected regions and their functional connectivity (Bestmann et al., 2005; Vink et al., 2018). Accumulating evidence suggests that the cerebellum is involved in language processing (Marien et al., 2014) and has reciprocal connections with key language regions such as the left inferior frontal gyrus and left lateral temporal gyrus (Booth et al., 2007). Furthermore, a study investigating functional connectivity revealed that the ATL was intrinsically coupled with the cerebellum (Jackson et al., 2016). Therefore, the observed cerebellum GM decrease following cTBS over the left ATL might reflect additional changes to functionally-connected remote areas. Alternatively, a local change of neural activity induced by rTMS can be integrated into whole brain dynamics to maintain global brain homoeostasis (Cocchi et al., 2015; Fornito et al., 2012). Studies combining fMRI with TMS have demonstrated that inhibitory rTMS/TBS can induce changes both to the targeted network and remote non-targeted systems, which balance the local neural specialization with large-scale dynamics (Cocchi et al., 2015; Jung et al., 2020). In line with these studies, the remote structural changes in the cerebellum observed in the current study, might be attributed to the local reorganization as a part of such whole brain dynamics.

In neuroimaging studies, small to moderate sample size can contribute to false positive findings (Button et al., 2013). Thus, our results should be interpreted cautiously, taking into account the small sample size. We performed a power calculation in this study to estimate TBS effects in behaviour: 2 factorial within subject design with site (ATL vs. Oz) x task (semantic vs. control). The estimated interaction effect between the site and task was 0.36 with 23 participants. To achieve α=0.05, power=80% for the critical interaction between site and task, *N* ≥ 18 are required. Although the power calculation showed that our sample size was sufficient to show the expected behavioural TBS effect, it remain unclear how many participants would be need to detect structural changes in VBM indices. With more subjects, we might see structural changes in the anterior commissure that we hypothesized as well as brain regions beyond the stimulation site. In addition, it should be noted that we only acquired the structural image after the stimulation without pre-stimulation scanning. Rather than the pre-stimulation scanning, we chose the control stimulation (Oz) as a baseline in this study. The Oz is commonly used as a control site in TMS literature. In our previous TMS semantic studies, Oz has successfully served as the control site, not influencing behavioural performance in either the semantic or control tasks (Lambon Ralph et al., 2009; Pobric et al., 2007, 2010a; Pobric et al., 2010b, 2009). Also, Oz stimulation did not cause any changes in the visual system in our fMRI data. To confirm our findings, future studies with pre and post stimulation scanning will be needed along with sham stimulation. Finally, although VBM has been widely used to detect brain morphological changes, this technique also has several limitations (please, see Mechelli et al., 2005 for the details): 1) VBM is sensitive to systematic shape differences attributable to misregistration; 2) It has difficulties in spatially normalizing and segmenting atypical brains that contains atypical tissue types, not present in the template image (GM, WM, and CSF); 3) The nature of grey and white matter changes identified with VBM is still poorly understood especially in healthy individuals. Therefore, our results should be carefully interpreted and further investigation will be needed to replicate these findings with larger sample size.

## Declaration of Competing Interest

The authors declare no competing financial interests.

## Appendix Supplementary materials

Image, application 1

## References

[bib0001] Agnew Z.K., Banissy M.J., McGettigan C., Walsh V., Scott S.K. (2018). Investigating the neural basis of theta burst stimulation to premotor cortex on emotional vocalization perception: a combined TMS-fMRI study. Front. Hum. Neurosci..

[bib0002] Allendorfer J.B., Storrs J.M., Szaflarski J.P. (2012). Changes in white matter integrity follow excitatory rTMS treatment of post-stroke aphasia. Restor. Neurol. Neurosci..

[bib0003] Andoh J., Paus T. (2011). Combining functional neuroimaging with off-line brain stimulation: modulation of task-related activity in language areas. J. Cogn. Neurosci..

[bib0004] Ashburner J., Friston K.J. (2000). Voxel-based morphometry–the methods. Neuroimage.

[bib0005] Benali A., Trippe J., Weiler E., Mix A., Petrasch-Parwez E., Girzalsky W., Eysel U.T., Erdmann R., Funke K. (2011). Theta-burst transcranial magnetic stimulation alters cortical inhibition. J. Neurosci..

[bib0006] Bestmann S., Baudewig J., Siebner H.R., Rothwell J.C., Frahm J. (2005). BOLD MRI responses to repetitive TMS over human dorsal premotor cortex. Neuroimage.

[bib0007] Binney R.J., Lambon Ralph M.A. (2015). Using a combination of fMRI and anterior temporal lobe rTMS to measure intrinsic and induced activation changes across the semantic cognition network. Neuropsychologia.

[bib0008] Booth J.R., Wood L., Lu D., Houk J.C., Bitan T. (2007). The role of the basal ganglia and cerebellum in language processing. Brain Res..

[bib0009] Bruel-Jungerman E., Davis S., Laroche S. (2007). Brain plasticity mechanisms and memory: a party of four. Neuroscientist.

[bib0010] Button K.S., Ioannidis J.P., Mokrysz C., Nosek B.A., Flint J., Robinson E.S., Munafo M.R. (2013). Power failure: why small sample size undermines the reliability of neuroscience. Nat. Rev. Neurosci..

[bib0011] Butz M., Worgotter F., van Ooyen A. (2009). Activity-dependent structural plasticity. Brain Res. Rev..

[bib0012] Chambers R.A., Potenza M.N., Hoffman R.E., Miranker W. (2004). Simulated apoptosis/neurogenesis regulates learning and memory capabilities of adaptive neural networks. Neuropsychopharmacology.

[bib0013] Cocchi L., Sale M.V., Lord A., Zalesky A., Breakspear M., Mattingley J.B. (2015). Dissociable effects of local inhibitory and excitatory theta-burst stimulation on large-scale brain dynamics. J. Neurophysiol..

[bib0014] Draganski B., Gaser C., Busch V., Schuierer G., Bogdahn U., May A. (2004). Neuroplasticity: changes in grey matter induced by training - Newly honed juggling skills show up as a transient feature on a brain-imaging scan. Nature.

[bib0015] Fornito A., Harrison B.J., Zalesky A., Simons J.S. (2012). Competitive and cooperative dynamics of large-scale brain functional networks supporting recollection. Proc. Natl. Acad. Sci. U. S. A..

[bib0016] Friston K.J., Harrison L., Penny W. (2003). Dynamic causal modelling. Neuroimage.

[bib0017] Funke K., Benali A. (2010). Cortical cellular actions of transcranial magnetic stimulation. Restor. Neurol. Neurosci..

[bib0018] Funke K., Benali A. (2011). Modulation of cortical inhibition by rTMS - findings obtained from animal models. J. Physiol.-Lond..

[bib0019] Good C.D., Johnsrude I.S., Ashburner J., Henson R.N., Friston K.J., Frackowiak R.S. (2001). A voxel-based morphometric study of ageing in 465 normal adult human brains. Neuroimage.

[bib0020] Halai A.D., Welbourne S.R., Embleton K., Parkes L.M. (2014). A comparison of dual gradient-echo and spin-echo fMRI of the inferior temporal lobe. Hum. Brain Mapp..

[bib0021] Hartwigsen G., Saur D., Price C.J., Ulmer S., Baumgaertner A., Siebner H.R. (2013). Perturbation of the left inferior frontal gyrus triggers adaptive plasticity in the right homologous area during speech production. Proc. Natl. Acad. Sci. U. S. A..

[bib0022] Holtmaat A., Svoboda K. (2009). Experience-dependent structural synaptic plasticity in the mammalian brain. Nat. Rev. Neurosci..

[bib0023] Huang Y.Z., Edwards M.J., Rounis E., Bhatia K.P., Rothwell J.C. (2005). Theta burst stimulation of the human motor cortex. Neuron.

[bib0024] Jackson R.L., Hoffman P., Pobric G., Lambon Ralph M.A. (2016). The Semantic Network at Work and Rest: differential Connectivity of Anterior Temporal Lobe Subregions. J. Neurosci..

[bib0025] Jung J., Bungert A., Bowtell R., Jackson S.R. (2020). Modulating brain networks with transcranial magnetic stimulation over the primary motor cortex: a concurrent TMS/fMRI study. Front Hum Neurosci.

[bib0026] Jung J., Lambon Ralph M.A. (2016). Mapping the dynamic network interactions underpinning cognition: a cTBS-fMRI study of the flexible adaptive neural system for semantics. Cereb. Cortex.

[bib0027] Kempermann G., Kuhn H.G., Gage F.H. (1997). More hippocampal neurons in adult mice living in an enriched environment. Nature.

[bib0028] Lambon Ralph M.A., Pobric G., Jefferies E. (2009). Conceptual knowledge is underpinned by the temporal pole bilaterally: convergent evidence from rTMS. Cereb. Cortex.

[bib0029] Lee L., Siebner H.R., Rowe J.B., Rizzo V., Rothwell J.C., Frackowiak R.S., Friston K.J. (2003). Acute remapping within the motor system induced by low-frequency repetitive transcranial magnetic stimulation. J. Neurosci..

[bib0030] Lefaucheur J.P., Andre-Obadia N., Antal A., Ayache S.S., Baeken C., Benninger D.H., Cantello R.M., Cincotta M., de Carvalho M., De Ridder D., Devanne H., Di Lazzaro V., Filipovic S.R., Hummel F.C., Jaaskelainen S.K., Kimiskidis V.K., Koch G., Langguth B., Nyffeler T., Oliviero A., Padberg F., Poulet E., Rossi S., Rossini P.M., Rothwell J.C., Schonfeldt-Lecuona C., Siebner H.R., Slotema C.W., Stagg C.J., Valls-Sole J., Ziemann U., Paulus W., Garcia-Larrea L. (2014). Evidence-based guidelines on the therapeutic use of repetitive transcranial magnetic stimulation (rTMS). Clin. Neurophysiol..

[bib0031] Lehner A., Langguth B., Poeppl T.B., Rupprecht R., Hajak G., Landgrebe M., Schecklmann M. (2014). Structural brain changes following left temporal low-frequency rTMS in patients with subjective tinnitus. Neural Plast..

[bib0032] Lenz M., Galanis C., Muller-Dahlhaus F., Opitz A., Wierenga C.J., Szabo G., Ziemann U., Deller T., Funke K., Vlachos A. (2016). Repetitive magnetic stimulation induces plasticity of inhibitory synapses. Nat. Commun..

[bib0033] Marien P., Ackermann H., Adamaszek M., Barwood C.H., Beaton A., Desmond J., De Witte E., Fawcett A.J., Hertrich I., Kuper M., Leggio M., Marvel C., Molinari M., Murdoch B.E., Nicolson R.I., Schmahmann J.D., Stoodley C.J., Thurling M., Timmann D., Wouters E., Ziegler W. (2014). Consensus paper: language and the cerebellum: an ongoing enigma. Cerebellum.

[bib0034] May A., Hajak G., Ganssbauer S., Steffens T., Langguth B., Kleinjung T., Eichhammer P. (2007). Structural brain alterations following 5 days of intervention: dynamic aspects of neuroplasticity. Cereb. Cortex.

[bib0035] Mechelli A., Price C.J., Friston K.J., Ashburner J. (2005). Voxel-based morphometry of the human brain: methods and applications. Curr. Med. Imaging Rev..

[bib0036] O'Shea J., Johansen-Berg H., Trief D., Gobel S., Rushworth M.F. (2007). Functionally specific reorganization in human premotor cortex. Neuron.

[bib0037] Oldfield R.C. (1971). The assessment and analysis of handedness: the Edinburgh inventory. Neuropsychologia.

[bib0038] Pekna M., Pekny M., Nilsson M. (2012). Modulation of Neural Plasticity as a Basis for Stroke Rehabilitation. Stroke.

[bib0039] Pell G.S., Roth Y., Zangen A. (2011). Modulation of cortical excitability induced by repetitive transcranial magnetic stimulation: influence of timing and geometrical parameters and underlying mechanisms. Prog. Neurobiol..

[bib0040] Pobric G., Jefferies E., Lambon Ralph M.A. (2007). Anterior temporal lobes mediate semantic representation: mimicking semantic dementia by using rTMS in normal participants. Proc. Natl. Acad. Sci. U. S. A..

[bib0041] Pobric G., Jefferies E., Lambon Ralph M.A. (2010). Category-specific versus category-general semantic impairment induced by transcranial magnetic stimulation. Curr. Biol..

[bib0042] Pobric G., Jefferies E., Ralph M.A. (2010). Amodal semantic representations depend on both anterior temporal lobes: evidence from repetitive transcranial magnetic stimulation. Neuropsychologia.

[bib0043] Pobric G., Lambon Ralph M.A., Jefferies E. (2009). The role of the anterior temporal lobes in the comprehension of concrete and abstract words: rTMS evidence. Cortex.

[bib0044] Sack A.T., Camprodon J.A., Pascual-Leone A., Goebel R. (2005). The dynamics of interhemispheric compensatory processes in mental imagery. Science.

[bib0045] Sagi Y., Tavor I., Hofstetter S., Tzur-Moryosef S., Blumenfeld-Katzir T., Assaf Y. (2012). Learning in the fast lane: new insights into neuroplasticity. Neuron.

[bib0046] Siebner H.R., Rothwell J. (2003). Transcranial magnetic stimulation: new insights into representational cortical plasticity. Exp. Brain Res..

[bib0047] Stephan K.E., Penny W.D., Daunizeau J., Moran R.J., Friston K.J. (2009). Bayesian model selection for group studies. Neuroimage.

[bib0048] Thomson A.C., Kenis G., Tielens S., de Graaf T., Schuhmann T., Rutten B.P.F., Sack A.T. (2020). Transcranial magnetic stimulation-induced plasticity mechanisms: tMS-related gene expression and morphology changes in a human neuron-like cell model. Front. Mol. Neurosci..

[bib0049] Trachtenberg J.T., Chen B.E., Knott G.W., Feng G.P., Sanes J.R., Welker E., Svoboda K. (2002). Long-term in vivo imaging of experience-dependent synaptic plasticity in adult cortex. Nature.

[bib0050] Trippe J., Mix A., Aydin-Abidin S., Funke K., Benali A. (2009). theta burst and conventional low-frequency rTMS differentially affect GABAergic neurotransmission in the rat cortex. Exp. Brain Res..

[bib0051] Valchev N., Gazzola V., Avenanti A., Keysers C. (2016). Primary somatosensory contribution to action observation brain activity-combining fMRI and cTBS. Soc Cogn Affect Neurosci.

[bib0052] Vink J.J.T., Mandija S., Petrov P.I., van den Berg C.A.T., Sommer I.E.C., Neggers S.F.W. (2018). A novel concurrent TMS-fMRI method to reveal propagation patterns of prefrontal magnetic brain stimulation. Hum. Brain Mapp..

[bib0053] Visser M., Jefferies E., Embleton K.V., Lambon Ralph M.A. (2012). Both the middle temporal gyrus and the ventral anterior temporal area are crucial for multimodal semantic processing: distortion-corrected fMRI evidence for a double gradient of information convergence in the temporal lobes. J. Cogn. Neurosci..

[bib0054] Xu T.H., Yu X.Z., Perlik A.J., Tobin W.F., Zweig J.A., Tennant K., Jones T., Zuo Y. (2009). Rapid formation and selective stabilization of synapses for enduring motor memories. Nature.

[bib0055] Zatorre R.J., Fields R.D., Johansen-Berg H. (2012). Plasticity in gray and white: neuroimaging changes in brain structure during learning. Nat. Neurosci..

